# Alleviation of Oral Exposure to Aflatoxin B1-Induced Renal Dysfunction, Oxidative Stress, and Cell Apoptosis in Mice Kidney by Curcumin

**DOI:** 10.3390/antiox11061082

**Published:** 2022-05-29

**Authors:** Yingjie Wang, Fangju Liu, Xin Zhou, Mengru Liu, Haoran Zang, Xiao Liu, Anshan Shan, Xingjun Feng

**Affiliations:** Laboratory of Molecular Nutrition, Institute of Animal Nutrition, Northeast Agricultural University, Harbin 150030, China; wangyingjie@neau.edu.cn (Y.W.); liufangju0701@163.com (F.L.); 18800430747@163.com (X.Z.); liumengruu@163.com (M.L.); zanghaoran811@163.com (H.Z.); liuxiao@neau.edu.cn (X.L.); asshan@neau.edu.cn (A.S.)

**Keywords:** aflatoxin B1, curcumin, kidney, oxidative stress, apoptosis

## Abstract

Aflatoxin B1 is a contaminant widely found in food and livestock feed, posing a major threat to human and animal health. Recently, much attention from the pharmaceutical and food industries has been focused on curcumin due to its strong antioxidant capacity. However, the therapeutic impacts and potential mechanisms of curcumin on kidney damage caused by AFB1 are still incomplete. In this study, AFB1 triggered renal injury in mice, as reflected by pathological changes and renal dysfunction. AFB1 induced renal oxidative stress and interfered with the Keap1–Nrf2 pathway and its downstream genes (CAT, SOD1, NQO1, GSS, GCLC, and GCLM), as manifested by elevated oxidative stress metabolites and reduced antioxidant enzymes activities. Additionally, AFB1 was found to increase apoptotic cells percentage in the kidney via the TUNEL assay, along with increased expression of Cyt-c, Bax, cleaved-Caspase-3, Caspase-9, and decreased expression of Bcl-2 at the transcriptional and protein levels; in contrast, for mice given curcumin, there was a significant reversal in kidney coefficient, biochemical parameters, pathological changes, and the expression of genes and proteins involved in oxidative stress and apoptosis. These results indicate that curcumin could antagonize oxidative stress and apoptosis to attenuate AFB1-induced kidney damage.

## 1. Introduction

Aflatoxins are mycotoxins generated by the filamentous fungi *Aspergillus flavus* and *Aspergillus parasiticus* [1]. They are unavoidable environmental contaminants and are widely found in human foods and animal feeds. Among the many recognized mycotoxins, aflatoxin B1 (AFB1) is the most toxic, carcinogenic, and teratogenic, and is vastly more harmful than organic pesticides, arsenicals, and cyanide [2,3]. Acceptable limits for AFB1 in foods consumed by people and animal feeds have been reported to be approximately parts per billion (ppb) and 300 ppb, respectively [4]. A high prevalence of AFB1 and its accumulation in humans and animals through the food chain can lead to serious health issues, including growth retardation, gastrointestinal and reproductive system diseases, tissue toxicity, neurotoxicity, and immunosuppression [5,6,7].

It is notable that the kidney is also an incipient target organ of AFB1 in addition to the liver [8]. Since metabolites of drugs or toxic substances are selectively absorbed and concentrated by renal tubular cells before being excreted in the urine, high concentrations of toxic substances are also deposited in the renal medulla [9,10]. This suggests that the kidney is also involved in the accumulation of toxin AFB1. Research has demonstrated that oxidative stress is a critical risk factor for AFB1 toxicity and that exposure to AFB1 raises contents of reactive oxygen species (ROS), which can impair cellular redox homeostasis, leading to oxidative stress-induced kidney injury [11]. Therefore, mitigation of oxidative stress is emphasized as an effective strategy to treat AFB1 nephrotoxicity.

Natural active substances derived from plants occupy a key position in research against aflatoxins [12,13]. Curcumin is a phenolic pigment extracted from the rhizome of *Curcuma longa* (turmeric) [14], which has been proven to exhibit multiple biological activities encompassing antioxidant, antibacterial, anti-inflammatory, and immune-enhancing properties in various experiments [15,16]. There is evidence that curcumin offers protective potential against tissue injury caused by certain drugs and environmental toxins: curcumin could prevent carbon tetrachloride (CCL4)-induced acute liver damage via inhibition of oxidative stress and inflammation [17]; curcumin can alleviate arsenic-induced hepatorenal toxicity by activating Nrf2 and inhibiting the MAPK–NF-κB pathway [18]; curcumin has also been found to exert cytoprotective and antioxidant effects against OTA-induced toxicity in rats [19]. These studies present evidence in support of the use of curcumin as a protective antioxidant. Moreover, curcumin has also been demonstrated to ameliorate AFB1-induced duodenal toxicity and liver injury by downregulating CYP450 enzyme activity and regulating hepatic long non-coding RNAs (LncRNAs) [20,21].

In our previous study, we also revealed that dietary curcumin could alleviate acute ileum damage by modulating NF-κB and Nrf2–ARE pathways in AFB1-treated Anas platyrhynchos. Nevertheless, few studies have been conducted on the protective effects of curcumin against AFB1-induced nephrotoxicity, mostly focusing on some apparent indicators, such as the administration of curcumin attenuated the oxidative stress parameters modified by AFB1 in chicken or mice kidneys and alleviated the structural damage of AFB1-induced renal cortex in rats [22,23,24]. However, the underlying mechanism remains unclear. Consequently, in this experiment, mice were administered with both AFB1 and curcumin via oral gavage at the same time, to explore the potential mechanisms of curcumin protection against kidney injury caused by AFB1 in terms of apparent growth, biochemical indices, pathological structure, ultrastructural observations, and expression of oxidative stress and apoptosis-related factors.

## 2. Materials and Methods

### 2.1. Animals and Experimental Design

In total, 50 Male BALB/c mice (5-week-old, 20–22 g body weight) were purchased from the Changsheng Biotechnology Co., Ltd. (Shenyang, China). Mice were housed in a controlled environment (22.5 ± 2 ℃ with 50–60% humidity) on a 12 h light–dark cycle. This animal experiment was approved by the Institutional Animal Care and Use Committee of Northeast Agricultural University (NEAU- [2011]- 9).

Mice were divided into 5 treatments randomly (n = 10) after acclimatization for one week. All mice were given oral gavage for 30 days: Control, received 0.2 mL olive oil as a vehicle; Cur200 and AFB1 groups were only administered 200 mg/kg bw curcumin (obtained from Sigma-Aldrich Ltd., Shanghai, China) and 750 μg/kg bw AFB1 (Sigma-Aldrich Ltd.), respectively; AFB1+ Cur100 and AFB1+ Cur200 were treated with 100 or 200 mg/kg bw of curcumin, respectively, while both were orally gavaged with 750 μg/kg bw AFB1. The AFB1 dosage was chosen according to previous studies [25,26]. AFB1, curcumin, or a mixture of both, were, respectively, administered in olive oil (0.2 mL) as a vehicle via oral gavage. In order to ensure uniformity of experimental conditions, the same volume of vehicle was administered to the mice. The health status of the mice was monitored daily throughout the experiment, and the dosage of curcumin and AFB1 was adjusted based on the weekly changes in body weight.

### 2.2. Sample Collection

All of the mice were fasting for 12 h before the end of the trial, and drinking water was normal. The obtained blood samples were centrifuged at 3500 rpm for 15 min to yield serum, which was stored at 80 °C until analysis. The kidney was resected and weighed. The renal index was calculated as kidney weight/mice weight × 100 [7]. A sample was taken from the left kidney, placed in 4% paraformaldehyde for H&E staining and TUNEL detection, and fixed in 2.5% glutaraldehyde to perform an ultrastructural inspection. The right kidney was kept at −80 °C in a freezer for the follow-up molecular assays.

### 2.3. Renal Function Determination

Commercially available kits (Nanjing Jiancheng Bioengineering Institute, Nanjing, China) were used for detecting the serum biochemical factors associated with kidney damage, such as uric acid (UA), creatinine (CREA), and urea nitrogen (BUN) using a Cobus-Mira-Plus automatic biochemical analyzer (Roche, Switzerland).

### 2.4. Hematoxylin–Eosin (H&E) Staining

After fixation of kidney tissues with 4% paraformaldehyde for 24 h, the samples were dehydrated, waxed, and embedded. The paraffin blocks were then cut into 5 µm thick sections by a microtome (Leica RM2016, Wetzlar, Germany) and stained with H&E. Renal histopathological damage was assessed using a Nikon Eclipse 80i microscope (Tokyo, Japan).

### 2.5. Transmission Electron Microscopy (TEM) Observations

The ultrastructure of tissue was observed according to the previous description [27], with some modifications. Briefly, each left kidney sample was cut into 1 mm^3^ pieces, fixed in 2.5% glutaraldehyde, and stored at 4 °C for 24 h under dark conditions. After rinsing with 0.1 M PBS, samples were then fixed in 1% osmic acid for 2 h and subsequently embedded in resin after dehydration with different concentrations of acetone. Next, the samples were sectioned using an ultramicrotome (Leica EM UC7, Wien, Austria), which were stained with uranyl acetate and lead citrate. Finally, ultrastructural images of the kidney tissue were observed via TEM (Hitachi HT7800, Hitachi, Japan).

### 2.6. TUNEL Fluorescence Staining to Detect the Rate of Apoptosis

The embedded mice kidney sections were deparaffinized and rehydrated according to the instructions of the TUNEL apoptosis assay kit (Beyotime, Shanghai, China). The sections were then incubated with a proteinase K working solution for 15–30 min at 37 °C. Subsequently, after washing three times in PBS, the TUNEL assay solution (green fluorescent probe fluorescein-labeled dUTP) was added dropwise to the samples and incubated (37 °C, 1 h). Staining and localization of cell nuclei were performed with DAPI. TUNEL-positive cells (green) were visualized with a fluorescence microscope (Olympus BX53, Tokyo, Japan), and the intensity of the fluorescent signal was analyzed with ImageJ software.

### 2.7. Antioxidant Levels in the Kidney

Kidney tissue samples were mixed with ice-cold saline (0.9% NaCl), homogenized, and centrifuged (4 °C, 900× *g*, 10 min), and the obtained supernatant was used for measuring redox indexes [7]. Beyotime bicinchoninic acid (BCA) determination kit was used to measure total protein concentration. The levels of hydrogen peroxide (H_2_O_2_), malondialdehyde (MDA), glutathione (GSH), and the activities of total antioxidant capacity (T-AOC), superoxide dismutase (SOD), and catalase (CAT) in the mice kidney were detected with commercial kits (Nanjing Jiancheng Bioengineering Institute, China).

### 2.8. Quantitative Real-Time PCR

Total RNA was prepared from mice kidney tissue using Trizol reagent (Takara, Dalian, China). RNA was reverse-transcribed into cDNA by a PrimeScript ™ RT reagent kit with a gDNA eraser (Takara) based on the manufacturer’s instructions. Synthesized cDNA was used for quantitative real-time polymerase chain reaction (Bio-Rad, Hercules, CA, USA) by SYBR premix EX Taq Master Mix (Takara) on Quantagene q225 (Kubo Tech, Beijing, China). Relative expressions of the genes were calculated with the 2^-ΔΔCt^ approach. All synthesized primer sequences are summarized in Table 1. β-actin was used as an internal standard for data analysis and normalization.

### 2.9. Western Blot Analysis

Western blot analysis was carried out, as described previously [28,29]. Briefly, proteins were extracted from kidney tissue samples using lysis buffer (Beyotime, Shanghai, China) containing 1 mM PMSF. The protein contents were then determined using a BCA kit. The protein samples were transferred onto PVDF membranes after separation on a 10% SDS–PAGE gradient gel. After blocking, these membranes were incubated overnight at 4 ℃ with the following primary antibodies at the stated dilutions: β-actin (1:1000, Beyotime,), Nrf2 (1:1000, Beyotime), Keap1 (1:1000, Beyotime), Bcl-2 (1:500, Wanleibio, Shenyang, China), Bax (1:500, Wanleibio), Cyt-c (1:500, Wanleibio) and cleaved Caspase-3 (1:500, Wanleibio). Membranes were incubated with secondary antibody (1:1000, Beyotime) conjugated to horseradish peroxidase after incubation with primary antibodies. The antigen–antibody complexes were visualized with a UVI gel documentation system (UVItec Ltd., Cambridge, UK). Relative intensities of these bands were quantified using ImageJ software and normalized to β-actin.

### 2.10. Statistical Analysis

The data were analyzed by one-way ANOVA and the least significant difference (LSD) tests. Data are displayed as mean ± standard error of the mean (SEM). Graphs were drawn using GraphPad Prism 8.3.0 (GraphPad Software, San Diego, CA, USA). Value of *p* < 0.05 or *p* < 0.01 was regard as statistically significant.

## 3. Results

### 3.1. Curcumin Improves Growth Retardation and Reduction in Renal Index in Mice Treated with AFB1

**Figure 1:**
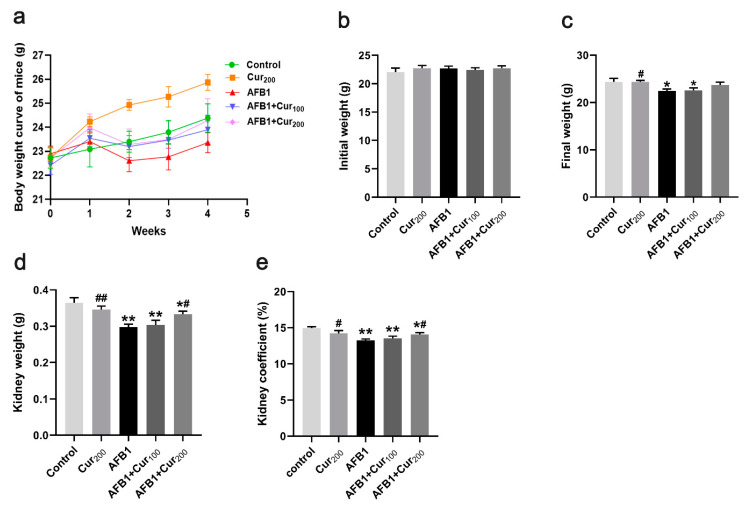
Body-weight changes and kidney index of mice: (**a**) Body weight of mice in each group at weeks 1, 2, 3, and 4; (**b**) initial weight; (**c**) final weight; (**d**) kidney weight; (**e**) kidney coefficient. All data are presented as mean ± SEM (n = 10). ‘*’ means significant difference (*p* < 0.05), and ‘**’ indicates extremely significant difference, compared with the control (*p* < 0.01); ‘#’ indicates a significant difference (*p* < 0.05), ‘##’ means a highly significant difference compared to AFB1 treatment (*p* < 0.01).

As depicted in Figure 1a,b, the initial body weight of mice did not differ significantly among treatments (*p* > 0.05). The growth curves showed that all mice gavaged with AFB1 showed a clear trend of weight loss at week 2, but the curcumin intervention slowed down the weight loss of mice in comparison to the group AFB1. From the 3rd week onwards, mice treated with AFB1 alone gradually gained weight, but it could be seen that their average body weights were consistently lower than that of the mice in other groups. Moreover, the mice in the control and Cur200 groups continued to gain weight during this test, with the Cur200 group showing a more pronounced increase. After one month trial, the final body weight, kidney weight, and kidney coefficient were significantly reduced in the AFB1, AFB1+Cur100, and AFB1+Cur200, compared with the control (*p* < 0.05, Figure 1c–e). However, in comparison with the group AFB1, the AFB1+Cur200 group significantly relieved AFB1-induced decrease in kidney weight and index (*p* < 0.05).

### 3.2. Curcumin Ameliorates AFB1-Induced Renal Dysfunction in Mice

**Figure 2:**
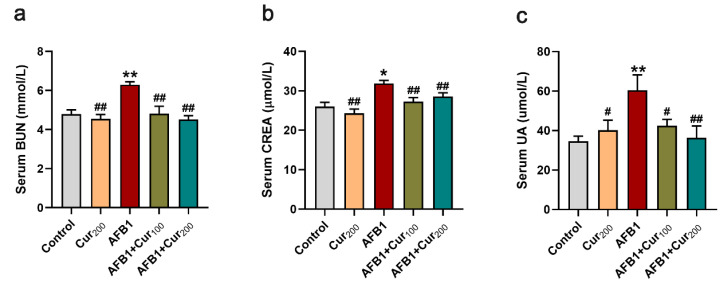
Detection results of serum biochemical parameters related to renal function in each group of mice: (**a**) blood urea nitrogen (BUN); (**b**) serum creatinine (CREA); (**c**) serum uric acid (UA). Results are presented as mean ± SEM (n = 10). ‘*’ and ‘**’ indicate significant difference (*p* < 0.05) or extremely significant difference (*p* < 0.01), respectively, compared with the control; ‘#’ demonstrates a significant difference (*p* < 0.05), ‘##’ means a highly significant difference compared with AFB1 treatment (*p* < 0.01).

Figure 2 illustrates the findings of BUN, CREA, and UA levels in mice serum. A highly significant increase in BUN, CREA, and UA content was observed in the AFB1 group versus the control (*p* < 0.05). However, the accumulation of BUN, CREA, and UA in mice kidneys in the curcumin intervention groups (AFB1+Cur100 and AFB1+Cur200) was significantly reduced compared with the AFB1-exposed group (*p* < 0.05). Based on these, it was concluded that curcumin could reduce the nephrotoxic damage in mice inflicted by AFB1.

### 3.3. Evaluation of Pathological Slices of the Kidney

**Figure 3:**
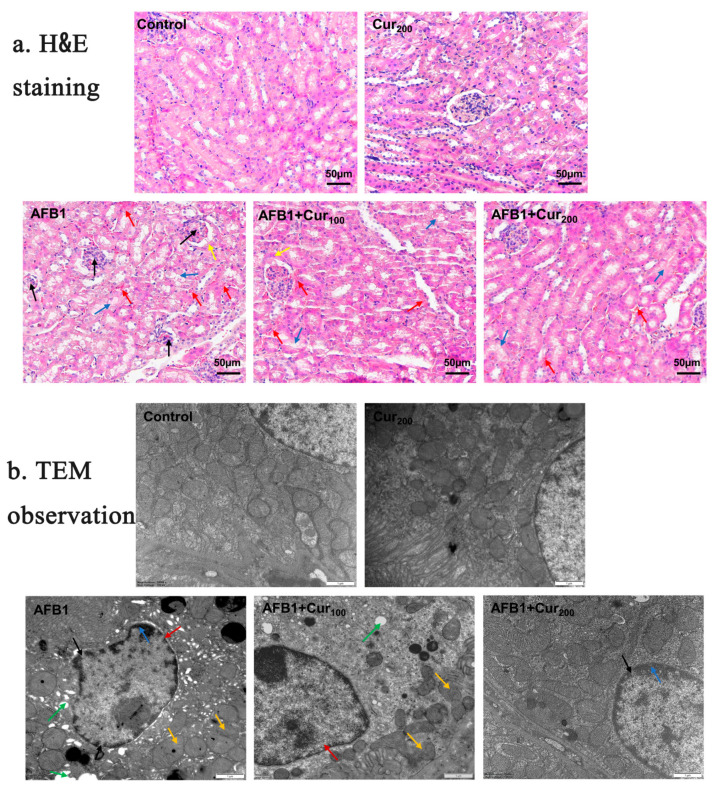
Influence of curcumin on pathological structure of kidney in mice subjected to AFB1 exposure: (**a**) changes in the renal slices (magnification × 200, scale bars = 50 μm). The arrows “→” represent pathological damage in the kidney. Black arrows indicate atrophy, and degeneration of the glomeruli; yellow arrows indicate swelling, and hyperplasia of the wall of the Bowman’s capsule; red arrows indicate congestion of the renal interstitium; blue arrows indicate detachment of renal tubular epithelial cells; (**b**) representative ultrastructure pictures of kidney in five groups (magnification× 20,000, scale bars = 1 μm). Black arrows indicate increased nuclear pores; green arrows show cytoplasmic vacuolation; blue arrows show chromatin margination; red arrows represent nuclear pyknosis and irregularities; yellow arrows indicate blurring or even disappearance of mitochondrial cristae.

Figure 3a shows H&E-stained sections of kidney tissues taken from each group of mice. No significant lesions were detected in the control and Cur200. Conversely, AFB1 treatment caused visible damage to kidney tissue, as reflected by glomerular atrophy or even degradation, swelling and hyperplasia of the Bowman’s capsule wall, degeneration and shedding of renal tubular epithelial cells, in addition to increased hemorrhagic spots in the renal interstitium and vacuolation-like changes in the renal tubules. Although hemorrhage and detachment of tubular epithelial cells were also seen in HE-stained sections of group AFB1+Cur100 and AFB1+Cur200, glomerular atrophy and tubular vacuolation-like lesions were significantly improved. This result confirmed that curcumin could somewhat reduce the histopathological lesions caused by AFB1 in the kidney of mice.

### 3.4. Ultrastructural Assessment of the Kidney

Changes in the ultrastructure of mice kidney cells were assessed using transmission electron microscopy (Figure 3b). Renal cells in the control and Cur200 groups exhibited normal nuclear morphology and intact mitochondrial structure. Severe vacuolization, nuclear membrane atrophy, increased nuclear pores, chromatin margination, and broken or even absent mitochondrial cristae were clearly observed in the AFB1 group. When curcumin was administered concomitantly with AFB1 exposure, nuclear pyknosis, chromatin condensation, and vacuolization were also noted, but the overall extent of lesions was clearly ameliorated compared to the AFB1 group. This result suggests that curcumin could improve the ultrastructure of the kidney impaired by AFB1.

### 3.5. Curcumin Ameliorates AFB1-Induced Kidney Oxidative Damage

**Figure 4:**
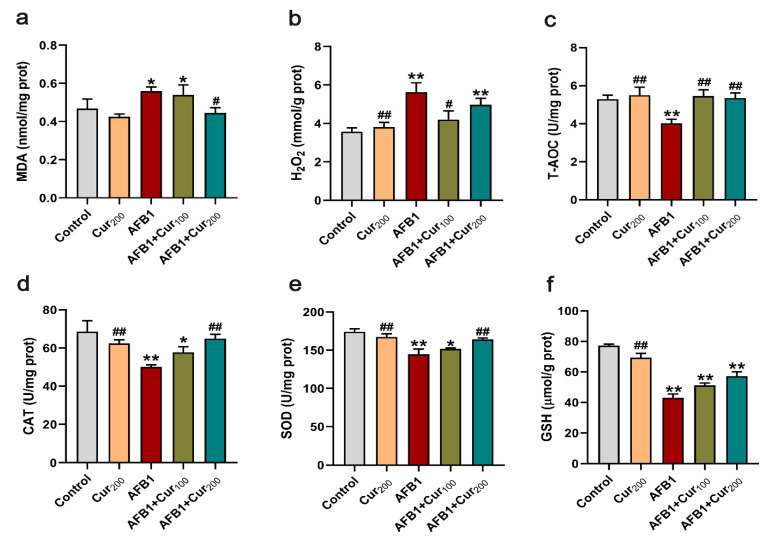
Evaluation of the antioxidant capacity of the kidney: (**a**,**b**) oxidative stress metabolites content: MDA and H_2_O_2_; (**c**) total antioxidant capacity: T-AOC; (**d**,**e**) antioxidant enzyme activities: CAT and SOD; (**f**) non-enzymatic antioxidant content: GSH. Data are presented as mean ± SEM (n = 8). ‘*’ means significant difference (*p* < 0.05), and ‘**’ indicates extremely significant difference compared with the control (*p* < 0.01); ‘#’ means a significant difference (*p* < 0.05), ‘##’ means a highly significant difference compared with the AFB1 group (*p* < 0.01).

To examine the redox status of mice kidneys, the contents of peroxides MDA and H_2_O_2_, T-AOC activity, the activities of antioxidant enzymes CAT and SOD, as well as the levels of non-enzymatic antioxidant GSH were measured. As presented in Figure 4, remarkable increases in the levels of oxidative stress metabolites MDA and H_2_O_2_ (*p* < 0.05) alongside substantial decreases in the T-AOC, CAT, and SOD activities and GSH concentration (*p* < 0.01) were noticed in mice kidneys in response to AFB1-induction, compared with the control. Contrastingly, curcumin treatment could reverse both oxidative stress markers and antioxidant enzyme alterations induced by AFB1 in mice kidneys. When compared with the AFB1 treatment, there was a significant decrease in MDA content of AFB1+Cur200 and H_2_O_2_ level of AFB1+Cur100, respectively (*p* < 0.05), while the activities of CAT, T-AOC, and SOD in AFB1+Cur200 were obviously upregulated (*p* < 0.05). Moreover, the two curcumin treatments also increased kidney GSH in mice to some extent, compared with the AFB1 group; however, the difference was not apparent (*p* > 0.05).

### 3.6. Curcumin Attenuates the AFB1-Induced Disturbance in Keap1–Nrf2 Antioxidant Signaling Pathway

**Figure 5:**
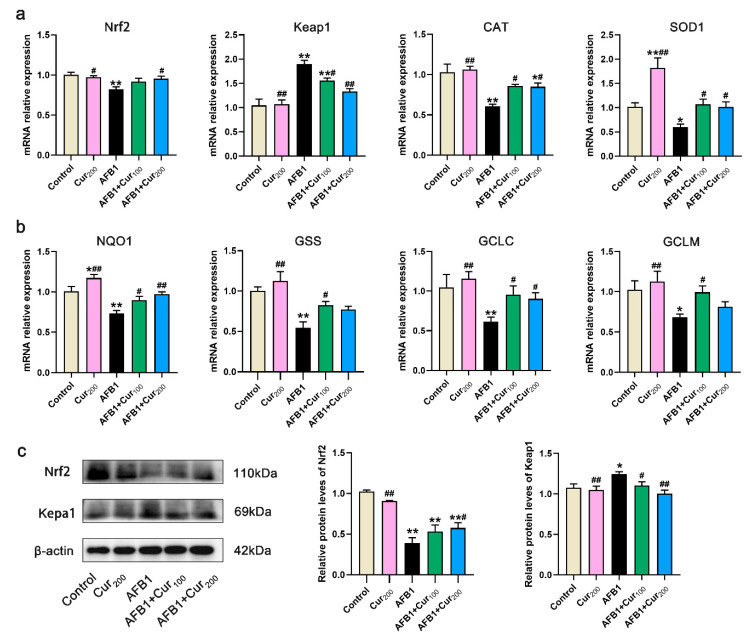
Gene and protein expressions associated with the Keap1–Nrf2 antioxidant pathway of the kidney: (**a**) the relative expression of the enzymatic antioxidant system Nrf2, Keap1, SOD, and CAT mRNA in the kidney was measured by RT-PCR; (**b**) relative mRNA expression levels of detoxification enzymes NQO1, GSS, GCLC and GCLM in the kidney; (**c**) protein expression of Nrf2 and Keap1 were examined by Western blot in renal tissue. Data are means ± SEM (n = 8). ‘*’ (*p* < 0.05) and ‘**’ (*p* < 0.01) indicates significant or highly significant differences, respectively, versus the control. ‘#’ (*p* < 0.05) and ‘##’ (*p* < 0.01) means difference or extremely difference, respectively, compared with the AFB1 treatment (*p* < 0.01).

As depicted in Figure 5a,b, compared with the control, the mRNA expression of genes for enzymatic antioxidant system (Nrf2, CAT, SOD1) and detoxifying enzymes (NQO1, GSS, GCLM, GCLC) in the Keap1–Nrf2 signaling pathway was dramatically lower in the AFB1-exposed group (*p* < 0.05), while the expression of Keap1, the repressor of Nrf2, was significantly higher (*p* < 0.01). Conversely, intervention groups with curcumin (AFB1+Cur100 and AFB1+Cur200) considerably improved the transcript expression of CAT, SOD1, NQO1, and GCLC in the Keap1–Nrf2 pathway disturbed by AFB1 (*p* < 0.05). Furthermore, examination of the expression for key proteins Nrf2 and Keap1 revealed a similar trend (Figure 5c). Although the protein expression level of Nrf2 was markedly downregulated (*p* < 0.01) in all AFB1-exposed groups, compared with the control, it was notably upregulated in the group AFB1+Cur200, compared with the AFB1 group (*p* < 0.05), and the expression of the Nrf2 blocker Keap1 was dramatically suppressed (*p* < 0.01). These findings indicate that curcumin can repair the disturbance of the Keap1–Nrf2 antioxidative pathway of mice kidneys by AFB1, as a means of reducing excessive oxidative damage.

### 3.7. Curcumin Protects against AFB1-Induced Renal Cell Apoptosis of Mice

**Figure 6:**
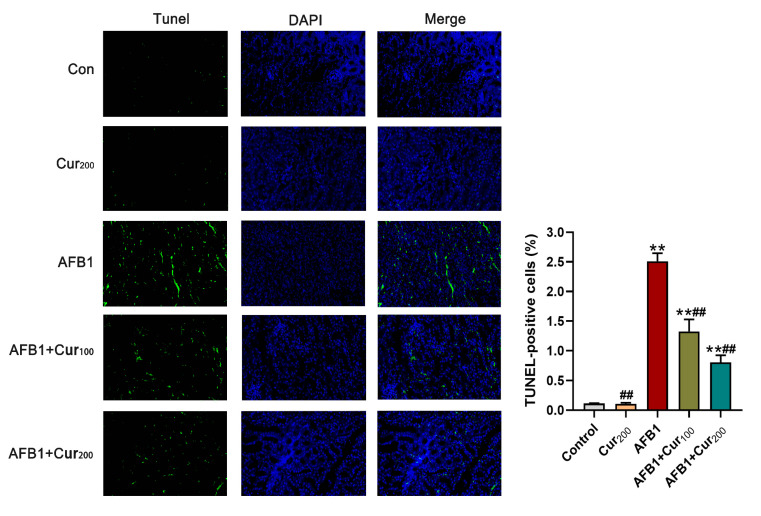
Terminal deoxynucleotidyl transferase dUTP nick end-labeling (TUNEL) staining results obtained for the mice kidney (magnification level = 200×). TUNEL (green) indicates apoptosis, DAPI (blue) locates the nucleus, and Merge is the combination of TUNEL and DAPI. ‘**’ means a highly significant difference (*p* < 0.01) versus the control. ‘##’ means an extremely difference, compared with AFB1 group (*p* < 0.01).

Figure 6 displays the results of the TUNEL staining assay in mice kidneys. In comparison with the control, the apoptosis rate of mice kidney cells was dramatically increased (*p* < 0.01) in groups AFB1, AFB1+Cur100, and AFB1+Cur200, but almost no variation in the Cur200 (*p* > 0.05). Apoptosis rates were obviously lower in curcumin-supplemented groups (AFB1+Cur100, AFB1+Cur200), compared with the AFB1 treatment (*p* < 0.01). This result shows that treatment with curcumin could decrease the number of apoptotic renal cells in mice exposed to AFB1.

### 3.8. Curcumin Restrains AFB1-Induced Mitochondrial Apoptosis Pathway in the Kidney

**Figure 7:**
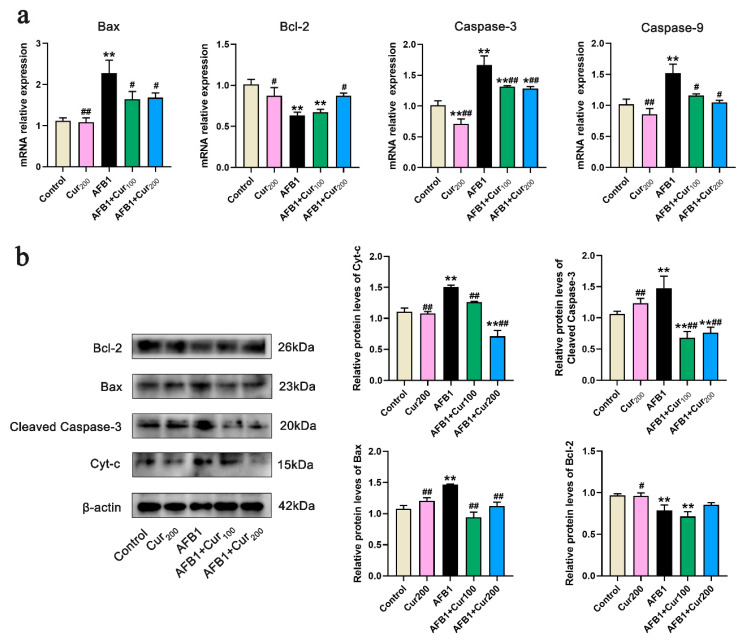
Gene and protein expression of apoptosis-related pathways in mice kidney (n = 8, mean with SEM). (**a**) relative mRNA expression levels of apoptosis-related genes Bax, Bcl-2, Caspase-3, and Caspase-9; (**b**) protein expression of apoptosis-related pathways, including Bcl-2, Bax, Cleaved Caspase-3 and Cyt-c. ‘*’ (*p* < 0.05) and ‘**’ (*p* < 0.01) represents significant or highly significant difference related to the control, repectively. ‘#’ (*p* < 0.05) and ‘##’ (*p* < 0.01) means difference or extremely difference, respectively, compared with the AFB1 group.

Based on the TUNEL results, we further examined apoptosis-related transcription and protein expression. As shown in Figure 7a, Bax, Caspase-3, and Caspase-9 transcript levels were remarkably increased (*p* < 0.01), whereas an observable reduction in Bcl-2 (*p* < 0.01) was observed in the mice kidney of the AFB1 challenge group, compared with the control. Conversely, compared with the AFB1 group, curcumin administration reversed the transcripts levels of these genes to some extent, with lower values for Bax, Caspase-9, and Caspase-3 relative expressions (*p* < 0.05), in both AFB1+Cur100 and AFB1+Cur200, and higher for Bcl-2 (*p* < 0.05) in the AFB1+Cur200 group.

Figure 7b displays the expression levels of apoptosis-related proteins. Compared with the control, there was a very pronounced increase in protein expression of Cyt-c, cleaved Caspase-3, and Bax (*p* < 0.01) in the kidney of mice from the AFB1 group, while Bcl-2 protein was strongly decreased (*p* < 0.01). However, in the groups of AFB1+Cur100 and AFB1+Cur200, Bax, Cyt-c, and cleaved Caspase-3 protein expressions were clearly downregulated (*p* < 0.01) with respect to the AFB1 group. Additionally, AFB1+Cur200 had a trend toward increased expression of the antiapoptotic protein Bcl-2 in comparison with the AFB1 exposure group but not significantly different (*p* > 0.05).

## 4. Discussion

AFB1 is the most toxic of many secondary metabolites produced by fungi, posing considerable health risks to people and animals due to its hepatotoxic, immunotoxic, carcinogenic, and teratogenic effects [30,31,32]. The role of oxidative stress associated with AFB1-mediated organ toxicity has been widely researched in animal models [11,33,34]. Among these studies, a great deal of attention has been devoted to the liver, which is the principal organ for AFB1 bioactivation. The kidney, on the other hand, has been relatively little studied as a vulnerable target for AFB1 following the liver. Furthermore, studies in experimental animal models strongly suggested that oxidative stress is a determinant of organ diseases triggered by AFB1 [35]. Polyphenolic curcumin, a natural antioxidant extracted from the rhizome of the turmeric plant, exhibits powerful antitoxic and antifungal effects [15,36]. In our previous study, curcumin was found to attenuate AFB1-induced liver injury [13]. Therefore, in order to assess the preventive impact of curcumin against AFB1-induced organ toxicity, the capacity of curcumin to counteract the effects of AFB1 on the kidney was further investigated.

In addition to the liver, the kidney is also considered to be an important target organ for the accumulation of AFB1 [37], as the compounds produced by AFB1 metabolism are mainly excreted through the kidney and the residual amount of toxin is higher in the kidney [38], so the toxicity of AFB1 to the kidney is not negligible. Based on the serological analysis showing the presence of higher concentrations of BUN, CREA, and UA after prolonged administration of AFB1, this leads to kidney damage that might involve inflammation, cell necrosis, and toxicosis [33,39]. Similarly, increased blood BUN and CREA as indicators of impaired renal function have been reported in broilers with AFB1 toxicity [40]. In our research, a pronounced increase in serum CREA, BUN, and UA concentrations was also noted after AFB1 exposure, and corresponding lesions were found in pathological sections and ultrastructural pictures, with assessments showing glomerular atrophy and swelling and hyperplasia of the Bowman’s capsule wall in the AFB1-treated group, as well as nuclear pyknosis and cytoplasmic vacuolization caused by AFB1. All these findings imply that AFB1 can cause nephrotoxicity, which is consistent with some previous findings. Salem et al. have reported renal congestion, vacuolization and tubular epithelial necrosis in chicks fed 0.25 ppm AFB1 [41]. Likewise, the main histopathological changes in the kidney of birds exposed to 0.5 ppm AFB1 including focal infiltration of inflammatory cells and glomerular atrophy were reported by Zabiulla et al. [42]. Weight loss is a marker of poisoning [43], and these nephrotoxic changes were also reflected in mice growth characteristics, as manifested by reduced body weight and kidney coefficients. It is noteworthy that body weight of mice in the AFB1 group showed a slow elevation in weeks 3 and 4, compared with the significant decrease in week 2, and the weight gain was similar in all AFB1-treated mice (including AFB1+Cur100 and AFB1+Cur200). The reason for this phenomenon may be that the mice entered the recovery phase after the AFB1 challenge, and their self-recovery capacity partially prevented the disturbance of the balance between orexigenic and anorexigenic circuits that regulate the homeostatic loop of body weight by AFB1, which is analogous to the results presented by Abdel-Wahhab et al. [44]. However, the body weight of mice in the AFB1-treated group was consistently the lowest among all groups. In contrast, curcumin treatment attenuated growth retardation and effectively blocked the AFB1-induced increases in BUN, CREA, and UA, as well as renal cell degeneration and necrosis. This suggests that curcumin may mitigate AFB1-induced renal injury from both physiological and pathological perspectives.

Oxidative stress can be triggered when the organism is exposed to external harmful stimuli, which leads to an increase in various ROS in the body, such as H_2_O_2_, superoxide anion (O_2_^•−^), and hydroxyl radicals (OH) [45,46]. AFB1 has been reported to increase the production of ROS [7]. Studies have demonstrated that biological macromolecules such as lipids, proteins, and nucleic acids could be damaged by excessive ROS, which generates massive amounts of MDA and induces tissue injury [47]. Therefore, lipid peroxide MDA is an important biomarker to assess oxidative damage. The T-AOC, GSH, CAT, and SOD in the endogenous antioxidant defense system could scavenge various ROS in the body and protect cells from oxidative damage [48]. SOD can convert superoxide radicals into H_2_O_2_, which is catalyzed by CAT and GSH into H_2_O and O_2_ [49,50]. In this study, exposure to AFB1 enhanced MDA and H_2_O_2_ levels in the kidney and significantly reduced the activity of T-AOC, CAT, GSH, and SOD. Apparently, AFB1 induced oxidative stress in mice kidneys. However, the curcumin intervention group significantly increased renal antioxidant enzyme activity, accompanied by reduced MDA and H_2_O_2_ levels, compared with the AFB1 group. In analogy to our results, a similar antioxidative effect of curcumin was also found in arsenic-treated mice kidneys, OTA -treated rat kidneys, and diesel exhaust particles (DEP)- and cisplatin (CP)-induced toxicity in human embryonic kidney cells (HEK-293) [19,36,51]. Consequently, the role of curcumin in protecting against oxidative stress in the kidney by reducing lipid peroxidation and increasing antioxidant status appears to be similar irrespective of host species.

The Keap1–Nrf2 signaling pathway is an efficacious approach for the body to alleviate stress, and it can resist endogenous or exogenous oxidative stress by encoding antioxidant enzymes and phase II detoxification enzymes [28]. Nrf2, an essential transcription factor for oxidative stress, is normally bound to its specific antagonist Keap in the cytoplasm, whereas when Nrf2 is stimulated by an oxidative stimulus such as ROS, it uncouples from Keap1 and enters the nucleus to bind to the ARE for regulating the downstream genes (SOD, CAT, GPx, NQO1, HO-1, GCLC, and GCLM) expression [52,53,54]. Here, a significant increase in Keap1 expression was noted after AFB1 was treated, while the expression of genes for Nrf2, the enzymatic antioxidant system (CAT, SOD), and phase II detoxifying enzymes (NQO1, GSS, GCLC, and GCLM) in the Keap1–Nrf2 pathway was reduced. Similarly, mRNA expression of Nrf2 and its downstream genes were significantly downregulated in the livers of broiler chickens and mice exposed to AFB1 (5 mg/kg diet and 750 μg/kg bw), respectively [26,55]. A supplementation of AFB1 (100 μg/kg) in the diet markedly diminished the protein expression of Nrf2 and HO-1 in chick bursa of Fabricius [56]. Nevertheless, with regard to the impacts of AFB1 on the Keap1–Nrf2 pathway, findings have been inconsistently reported previously. Liu and Wang observed that AFB1 exposure at 0.5–5 μmol/L notably upregulated Nrf2 expression in primary broiler hepatocytes (PBHs) [57]. Analogous results have also been reported in broiler cardiomyocytes (BCMs) [58]. The reason for this phenomenon may be due to the possible dual role of Keap1–Nrf2 in cells, which is activated by low levels of AFB1 stimulation to protect cells from oxidative damage, whereas high doses of AFB1 beyond its tolerance range may trigger excessive oxidative stress, thereby downregulating the expression of genes related to this pathway. This also confirms the involvement of the Keap1–Nrf2 family in AFB1-induced nephrotoxicity in mice in vivo.

In addition, as a natural plant antioxidant, curcumin potently activated Nrf2 to prevent hepatorenal injury induced by arsenic overdose and dimethylnitrosamine toxicity [18,59]. Similar to our results, we found that the administration of AFB1 and curcumin promoted the expression of Nrf2 and its downstream genes that were inhibited by AFB1 in the kidney. This suggests that curcumin can enhance renal antioxidant capacity and reduce redox stress caused by AFB1 by activating the Nrf2 signal pathway. Furthermore, it is worth noting that we found no noticeable changes in the Nrf2 gene and protein expression in the group treated with curcumin alone, compared with the control, suggesting that curcumin may not cause significant Nrf2 translocation in the absence of external stress stimuli.

Apoptosis is a key physiological mechanism that can be activated by oxidative stress [34]. AFB1 has been reported to induce liver DNA damage in the animal body, increasing MDA and ROS levels, which, in turn, leads to cell apoptosis [7]. In our experiment, the TUNEL staining result revealed that AFB1 greatly increased the apoptosis rate of mice kidney cells, which is consistent with an increase in oxidation products (MDA and H_2_O_2_) in the kidney. This also confirms that renal apoptosis is caused by AFB1-mediated oxidative stress. Research has shown that the primary target of the deleterious effects of AFB1 leading to apoptosis is the mitochondria [2]. We, therefore, further analyzed the expression of genes and proteins involved in the mitochondrial apoptotic pathway in the kidney.

Changes in the expression of proapoptotic Bax and antiapoptotic Bcl-2 govern the mitochondria-mediated apoptotic pathway [60]. As a proapoptotic protein, Bax induces cell apoptosis by competing with Bcl-2 to promote Cyt-c release into the cytoplasm, leading to Caspase-9/3 cascade activation [61]. Specifically, activated Caspase-9 can directly induce caspase-3 to an active cleaved form (cleaved Caspase-3) and trigger mitochondria-mediated apoptosis [62]. Therefore, Caspase-9 is generally considered to be a biomarker of the mitochondrial apoptotic pathway, while cleaved Caspase-3 is a critical enforcer of apoptosis. Our results revealed that AFB1 significantly upregulated the expression of Bax, Cyt-c, Caspase-3, and Caspase-9, while Bcl-2 was downregulated. In addition to our results, others have also shown that AFB1 induces cell apoptosis in different tissues (such as the thymus, spleen, liver, and testis) through a highly reactive oxygen-radical-mediated mitochondrial pathway [2,25,34,63].

Apoptosis is one of the histotoxic mechanisms of mycotoxins [59]. Since AFB1 can cause renal apoptosis, attenuating apoptosis can reduce AFB1 toxicity. In this assay, TUNEL staining results showed that curcumin treatment remarkably decreased AFB1-induced apoptosis in kidney cells. Moreover, curcumin treatment promoted Bcl-2 expression and inhibited the expression of Bax, Cyt-c, and cleaved Caspase-3 protein in the kidney of mice under AFB1 exposure. This suggests that curcumin can reduce AFB1-induced renal apoptosis via the mitochondrial pathway, thereby alleviating AFB1-induced nephrotoxicity.

## 5. Conclusions

**Figure 8:**
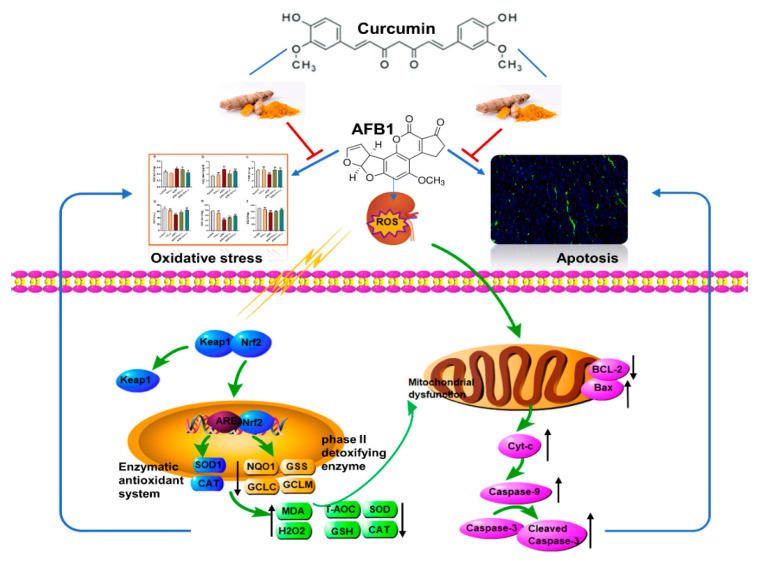
Main mechanism of curcumin in protecting against kidney oxidative damage and cell apoptosis caused by aflatoxin B1.

In summary, we proved that orally administered curcumin in mice could antagonize the deleterious effects of AFB1 on the kidney, and this effect is realized by modulating the Keap1–Nrf2 signal pathway to enhance the renal antioxidant capacity and inhibiting Bax/Bcl-2–Cyt-c signaling cascade-mediated apoptosis (Figure 8). The results of this trial deepen our knowledge of the role of curcumin in ameliorating AFB1-induced renal injury and support the application of curcumin as a dietary additive against AFB1 toxicity.

## Figures and Tables

**Table 1:** Primers used in the gene expression analysis.

Transcripts	Primer Sequence (5′-3′)	Product Size (bp)	Accession No.
Keap1	F: GACTGGGTCAAATACGACTGC	165	NM_001110307.1
	R: GAATATCTGCACCAGGTAGTCC	165	
Nrf2	F: AAGCACAGCCAGCACATTCTCC	130	NM_010902.4
	R: TGACCAGGACTCACGGGAACTTC	130	
SOD1	F: TGTCCATTGAAGATCGTGTGAT	85	NM_011434.1
	R: TCATCTTGTTTCTCATGGACCA	85	
GCLC	F: CTATCTGCCCAATTGTTATGGC	120	NM_010295.2
	R: CCTCCCGTGTTCTATCATCTAC	120	
GCLM	F: CTTGGAGCATTTACAGCCTTAC	226	NM_008129.4
	R: GTGAGTCAGTAGCTGTATGTCA	226	
GSS	F: CTGATGCTAGAGAGATCTCGTG	186	NM_001291111.1
	R: TTCACCCATGTCCAGTGAATAG	186	
CAT	F: CACCTTCAAGTTGGTTAATGCA	199	NM_009804.2
	R: CATGACCTGGATGTAAAACGTC	199	
NQO-1	F: GAAGACATCATTCAACTACGCC	179	NM_008706.5
	R: GAGATGACTCGGAAGGATACTG	179	
Caspase-3	F: GAAACTCTTCATCATTCAGGCC	250	NM_010295.2
	R: GCGAGTGAGAATGTGCATAAAT	250	
Caspase-9	F: TGTGAATATCTTCAACGGGAGC	249	NM_001277932.1
	R: GAGTAGGACACAAGGATGTCAC	249	
Bax	F: TTGCCCTCTTCTACTTTGCTAG	81	NM_007527.3
	R: CCATGATGGTTCTGATCAGCTC	81	
Bcl-2	F: GATGACTTCTCTCGTCGCTAC	156	NM_009741.5
	R: GAACTCAAAGAAGGCCACAATC	156	
β-actin	F: CTACCTCATGAAGATCCTGACC	90	NM_007393.5
	R: CACAGCTTCTCTTTGATGTCAC	90	

## Data Availability

The data is contained within the article.
